# The Rise of Borderline Personality Disorder in Kyrgyzstan: A Two-Year Review

**DOI:** 10.1192/j.eurpsy.2025.1924

**Published:** 2025-08-26

**Authors:** E. Molchanova

**Affiliations:** 1 Psychology, American University in Central Asia, Bishkek, Kyrgyzstan

## Abstract

**Introduction:**

Borderline Personality Disorder (BPD) was historically an underdiagnosed condition in Kyrgyzstan, often identified only in extreme cases, such as when individuals engaged in self-harm or displayed severe emotional instability. Traditionally, psychiatric consultations for BPD were initiated not by the individuals themselves but by concerned family members, typically parents. As mental health was heavily stigmatized in Kyrgyz society, especially personality disorders, many individuals likely went undiagnosed or untreated.

From 2020 to 2022, the Institute of Behavioral Health at the American University of Central Asia (AUCA) identified only 15 cases of BPD, However, since 2022, there has been a significant rise in BPD diagnoses, with over 70 cases recorded in just two years.

**Objectives:**

This study aims to examine the factors contributing to the increase in BPD diagnoses in the Kyrgyz Republic over the past two years.

**Methods:**

The study’s methodology includes the analysis of secondary data provided by mental health organizations, including the AUCA Institute of Behavioral Health, which has tracked BPD cases in recent years. Additionally, anecdotal reports from practicing clinicians offer insights into the evolving nature of mental health diagnoses and treatments in Kyrgyzstan.

**Results:**

Several key factors have contributed to the rise in BPD diagnoses in Kyrgyzstan over the past two years. First, socio-economic stressors have intensified, particularly following the political and economic challenges of 2022. Financial instability and high unemployment rates have exacerbated psychological stress for many individuals. These conditions often worsen emotional dysregulation, a core feature of BPD, particularly for those already predisposed to the disorder. Second, the proliferation of social media has played a notable role in shaping mental health patterns, especially among young people. Increased social media exposure has been linked to feelings of inadequacy, identity confusion, and emotional instability—all key components of BPD. Third, domestic violence and trauma remain significant public health concerns in Kyrgyzstan. BPD has long been associated with adverse childhood experiences, including emotional neglect, sexual abuse, and physical violence, making trauma a significant factor in the development of the disorder. As trauma rates rise, so too does the likelihood of developing BPD, especially in those with preexisting vulnerabilities. Moreover, the reduction in stigma surrounding mental health in recent years has also contributed to the rise in diagnoses. Consequently, mental health professionals are now diagnosing BPD earlier and more frequently than in previous years.

**Conclusions:**

The sharp increase in BPD cases in Kyrgyzstan can be attributed to a combination of socio-economic, psychological, and cultural shifts. Addressing underlying socio-economic and trauma-related factors remains crucial.

**Disclosure of Interest:**

None Declared

